# The first structural characterization of the proton­ated aza­cyclam ligand in *catena*-poly[[[(perchlorato)copper(II)]-μ-3-(3-carb­oxy­prop­yl)-1,5,8,12-tetra­aza-3-azonia­cyclo­tetra­deca­ne] bis­(per­chlorate)]

**DOI:** 10.1107/S205698901901377X

**Published:** 2019-10-22

**Authors:** Liudmyla V. Tsymbal, Vladimir B. Arion, Yaroslaw D. Lampeka

**Affiliations:** a L.V. Pisarzhevskii Institute of Physical Chemistry of the National Academy of Sciences of Ukraine, Prospekt Nauki 31, Kiev 03028, Ukraine; b Institute of Inorganic Chemistry of the University of Vienna, Wahringer Str. 42, 1090 Vienna, Austria

**Keywords:** crystal structure, aza­macrocyclic ligand, aza­cyclam, copper, coordination polymer, hydrogen bonds

## Abstract

The asymmetric unit of the title com­pound, *catena*-poly[[[(perchlorato-κ*O*)copper(II)]-μ-3-(3-carb­oxy­prop­yl)-1,5,8,12-tetra­aza-3-azonia­cyclo­tetra­decane-κ^4^ *N* ^1^,*N* ^5^,*N* ^8^,*N* ^12^] bis­(per­chlorate)], {[Cu(C_13_H_30_N_5_O_2_)(ClO_4_)](ClO_4_)_2_}_*n*_, (I), consists of a macrocyclic cation, one coordinated per­chlorate anion and two per­chlorate ions as counter-anions. The metal ion is coordinated in a tetra­gonally distorted octa­hedral geometry by the four secondary N atoms of the macrocyclic ligand, the mutually *trans* O atoms of the per­chlorate anion and the carbonyl O atom of the protonated carb­oxy­lic acid group of a neighbouring cation. The average equatorial Cu—N bond lengths [2.01 (6) Å] are significantly shorter than the axial Cu—O bond lengths [2.379 (8) Å for carboxyl­ate and average 2.62 (7) Å for disordered per­chlorate]. The coordinated macrocyclic ligand in (I) adopts the most energetically favourable *trans*-III conformation with an equatorial orientation of the substituent at the protonated distal 3-position N atom in a six-membered chelate ring. The coordination of the carb­oxy­lic acid group of the cation to a neighbouring com­plex unit results in the formation of infinite chains running along the *b*-axis direction, which are cross­linked by N—H⋯O hydrogen bonds between the secondary amine groups of the macrocycle and O atoms of the per­chlorate counter-anions to form sheets lying parallel to the (001) plane. Additionally, the extended structure of (I) is consolidated by numerous intra- and interchain C—H⋯O contacts.

## Abstract 2

The title com­pound is one-dimensional coordination polymer built up of tetra­gonally distorted CuN_4_O_2_ octa­hedra formed by four N atoms of the aza­macrocyclic ligand in the equatorial plane and O atoms of the protonated carb­oxy­lic acid group and the per­chlorate anion in the axial positions. In the crystal, [010] polymeric chains are crosslinked by N—H⋯O hydrogen bonds to form sheets lying parallel to the (001) plane.

## Chemical context   

Because of their exceptionally high thermodynamic stability and kinetic inertness (Melson, 1979 ▸; Yatsimirskii & Lampeka, 1985 ▸), transition-metal com­plexes of the macrocycles 1,4,8,11-tetra­aza­cyclo­tetra­decane (cyclam), *N* ^3^,*N* ^10^-disubstituted 1,3,5,8,10,12-hexa­aza­cyclo­tetra­decane (di­aza­cyclam) and, to a lesser extent, *N* ^3^-substituted 1,3,5,8,12-penta­aza­cyclo­tetra­decane (aza­cyclam) are popular building units for the assembly of metal–organic frameworks (MOFs), demonstrating many promising applications (Lampeka & Tsymbal, 2004 ▸; Suh & Moon, 2007 ▸; Suh *et al.*, 2012 ▸; Stackhouse & Ma, 2018 ▸). Two latter types of the Cu^II^ and Ni^II^ com­plexes are readily obtainable *via* template-directed Mannich condensation of bis­(ethyl­enedi­amine) or 3,7-di­aza­nonane-1,9-di­amine com­plexes, respectively, with formaldehyde and primary amines (Rosokha *et al.*, 1993 ▸; Costisor & Linert, 2000 ▸). The use of primary amines bearing a carb­oxy­lic acid function as locking fragments in these template reactions allows for the preparation of com­plexes of carboxyl-functionalized di­aza­cyclams, as was shown for the Ni^II^ and Cu^II^ com­plexes of di­aza­cyclam substituted with 3-carb­oxy­propyl groups (Lu *et al.*, 2005 ▸; Ou *et al.*, 2005 ▸). Such com­pounds are of particular inter­est because they can self-polymerize due to the coordination of the donor group of the substituent to the metal ion of another mol­ecule, thus forming coordination polymers without using additional bridging ligands, the most popular of which are carboxyl­ates (Rao *et al.*, 2004 ▸). Indeed, the Cu^II^ com­plex of this di­aza­cyclam ligand possesses a self-polymeric structure (Ou *et al.*, 2005 ▸), whereas the Ni^II^ complex does not form a polymer (Lu *et al.*, 2005 ▸). Data on the polymeric com­pounds of the given type formed by the com­plexes of functionalized aza­cyclam are not available in the literature so far.
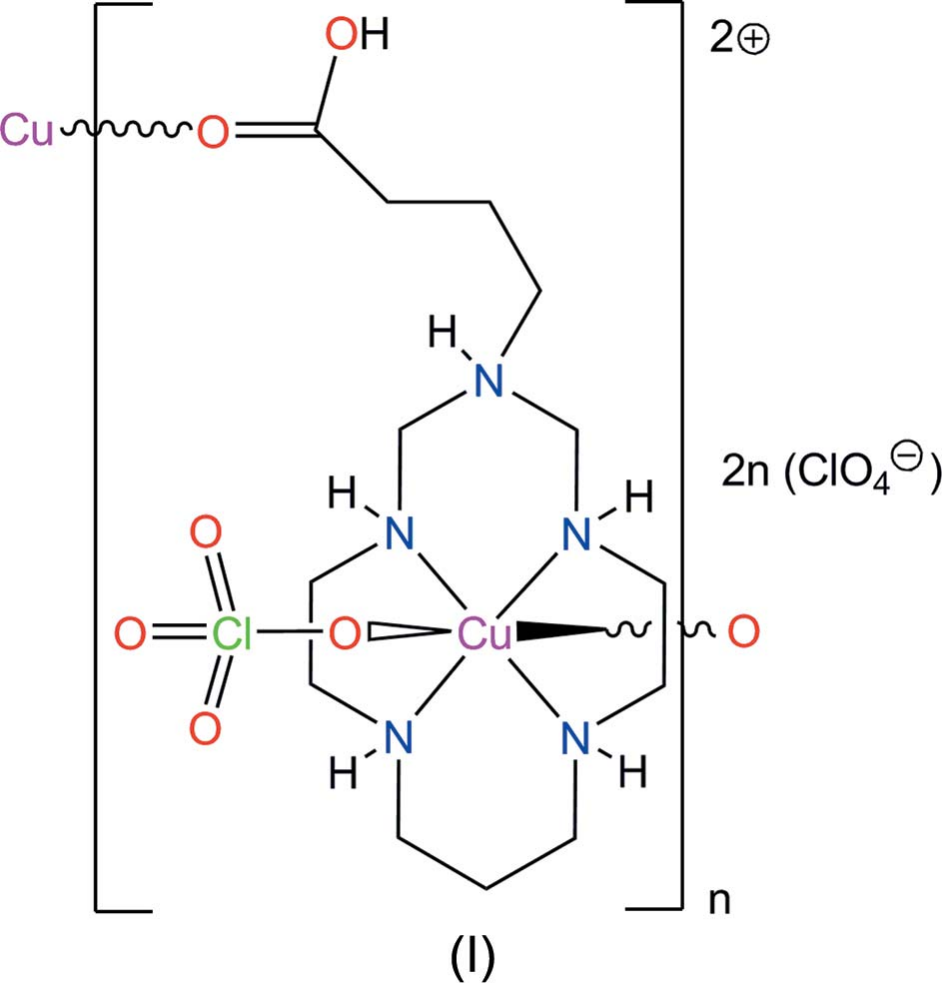


Another issue of inter­est is the acid–base properties of the non­coordinated distal N atom present in the macrocyclic backbones of aza- and di­aza­cyclams. Its likely proton­ation was postulated first based on the solution properties of the Ni^II^ com­pounds (Rosokha *et al.*, 1993 ▸; Tsymbal *et al.*, 1995 ▸; Hay *et al.*, 1997 ▸) and was further confirmed by X-ray structural analysis of the diethyl-substituted Ni^II^ di­aza­cyclam com­plex (Jiang *et al.*, 2006 ▸), while such a possibility for the Cu^II^ com­plexes has not been reported yet.

Herein, we describe the synthesis and the crystal structure of the title Cu^II^ com­plex, (I), with a protonated aza­cyclam ligand bearing a carb­oxy­lic acid group, namely, *catena*-poly[[[(perchlorato-κ*O*)copper(II)]-μ-3-(3-carb­oxy­prop­yl)-1,5,8,12-tetra­aza-3-azonia­cyclo­tetra­decane-κ^4^ *N* ^1^,*N* ^5^,*N* ^8^,*N* ^12^] bis­(per­chlorate)], {[Cu(H_2_ *L*)(ClO_4_)](ClO_4_)_2_}_*n*_, which is the first example of aza­cyclam ligand with a carb­oxy­lic acid group.

## Structural commentary   

**Figure 1:**
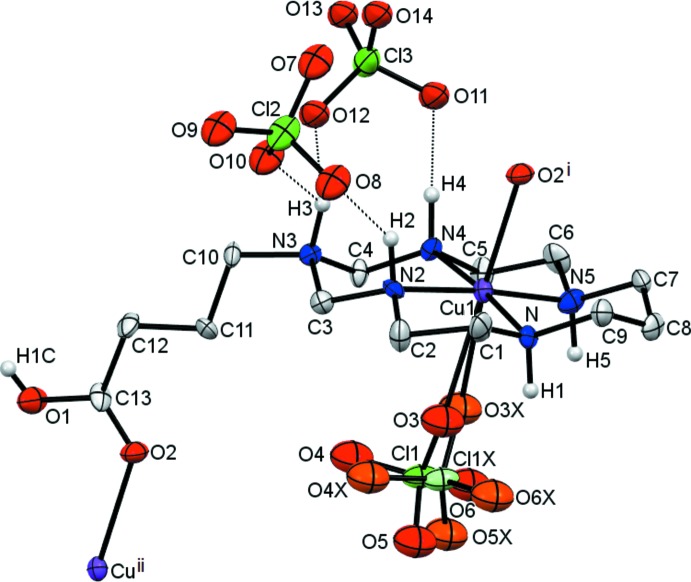
View of the asymmetric unit of (I), expanded to show the linking atoms O2^i^ and Cu1^ii^ forming the [010] polymeric chains, with displacement ellipsoids drawn at the 30% probability level. H atoms attached to C atoms have been omitted for clarity. The coordinated per­chlorate anion (Cl1O_4_) is equally disordered over two sets of sites and is shown in different shades; the minor-disorder com­ponents of the per­chlorate counter-anions with site occupancies of 20 (Cl2O_4_) and 22% (Cl3O_4_) have been omitted for clarity. Dashed lines represent hydrogen bonds. [Symmetry codes: (i) *x*, *y* + 1, *z*; (ii) *x*, *y* − 1, *z*.]

The Cu^II^ ion in the com­plex cation in (I) is coordinated by four secondary amine N atoms of the aza­macrocyclic ligand in a square-planar fashion and by O atoms from the per­chlorate anion and the carb­oxy­lic acid group of a neighbouring cation in the axial positions, resulting in a tetra­gonally distorted octa­hedral geometry (Table 1 ▸ and Fig. 1 ▸). The Cu^II^ ion is displaced by 0.075 Å from the mean plane of the N_4_ donor atoms (r.m.s. deviation = 0.005 Å) towards the O2 atom of the carboxyl­ate group. The equatorial Cu—N bond lengths are significantly shorter than the axial Cu—O bond lengths (Table 1 ▸), which can be attributed to a large Jahn–Teller distortion.

The macrocyclic ligand in (I) adopts the most energetically favourable *trans*-III (*R*,*R*,*S*,*S*) conformation (Bosnich *et al.*, 1965 ▸), with the five-membered chelate rings in *gauche* [average bite angle = 86.2 (18)°] and the six-membered chelate rings in *chair* [average bite angle = 93.6 (2)°] conformations. The methyl­ene group of the substituent at the noncoordinated N3 atom in the six-membered chelate ring is oriented equatorially. Such an arrangement of the substituent, in contrast to an axial orientation, is relatively uncommon and only a few examples of such Cu^II^ com­plexes with aza- and di­aza­cyclam ligands have been described so far (Shin *et al.*, 2010 ▸, 2012 ▸; Tsymbal *et al.*, 2010 ▸; Husain *et al.*, 2012 ▸; Xia *et al.*, 2014 ▸).

The formation of the azonia N3H^+^ group in (I) leads to clear changes in the C—N—C angles com­pared to the nonprotonated ones. The sum of these angles in the latter case (345–354°) is much larger than the canonical value for an *sp* ^3^-hybridized N atom (*ca* 327°), thus indicating their partial *sp* ^2^ character (Tsymbal *et al.*, 2010 ▸; Andriichuk *et al.*, 2019 ▸), while in (I) this parameter equals 335 (2)°, demonstrating an *sp* ^2^-to-*sp* ^3^ transformation of the noncoordinated N atom upon protonation.

The C—O bond lengths in the carb­oxy­lic acid group of the substituent differ considerably [1.318 (13) and 1.198 (13) Å for C13—O1 and C13—O2, respectively], thus confirming its protonated form and the lack of delocalization. Inter­estingly, it is coordinated to the Cu^II^ ion *via* O2, the carbonyl O atom, which is analogous to the situation observed in a bis­(3-carb­oxy­prop­yl)-substituted di­aza­cyclam polymeric com­plex (Ou *et al.*, 2005 ▸).

Three disordered per­chlorate anions in the title com­pound counterbalance the charge of the com­plex cations. The Cl1O_4_ anion is com­pletely disordered over two positions with site occupancies of 50% and is weakly coordinated to the metal ion (Table 1 ▸). Two remaining counter-anions are partially disordered with the retention of the positions of the central Cl atoms, with site occupancies of the major com­ponents of 80 (Cl2O_4_) and 78% (Cl3O_4_). Because of the low partial population, the minor com­ponents of these per­chlorate anions were not considered further in the analysis of the hydrogen-bonding network.

## Supra­molecular features   

**Figure 2:**
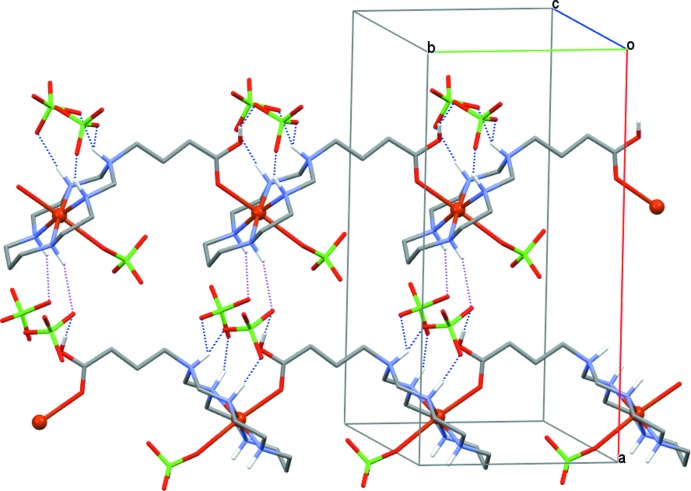
The packing in (I), showing [010] polymeric chains crosslinked by N—H⋯O hydrogen bonds to form sheets lying parallel to the (001) plane. H atoms at C atoms of the macrocyclic ligands have been omitted, as has one disorder com­ponent of the per­chlorate anions coordinated to Cu^II^ and the minor-disorder com­ponents of the non­coordinated per­chlorate counter-anions. Intra- (blue) and inter­chain (purple) N—H⋯O hydrogen bonds are shown as dashed lines.

The inter-cationic coordination of the carb­oxy­lic acid group of the substituent in the macrocycle to the metal ion results in the formation of one-dimensional polymeric chains running along the *b*-axis direction (Fig. 2 ▸). These chains are further reinforced by hydrogen bonding between secondary amine groups of the macrocycle acting as proton donors and O atoms of the per­chlorate anions as proton acceptors [N2—H2⋯O8(Cl2) and N4—H4⋯O11(Cl3)]. Additionally, the azonia group of the macrocycle forms a bifurcated hydrogen bond with both non­coordinated per­chlorate anions [N3—H3^+^⋯O10(Cl2),O12(Cl3)], so that each per­chlorate anion is fixed in a chain in a ditopic manner (Fig. 2 ▸ and Table 2 ▸). In addition, weak hydrogen bonding exists between the carb­oxy­lic acid group as the proton donor and an O atom of one of the per­chlorate ions [O1—H1*C*⋯O7(Cl2)(*x*, *y* − 1, *z*)], as well as between secondary amine groups of the macrocycle and an O atom of the carb­oxy­lic acid group as the proton acceptor [N2—H2(N4—H4)⋯O1(*x*, *y* + 1, *z*)]. Hydrogen bonding of the secondary amine groups of the macrocycle and the O atoms of per­chlorate anions not involved in above-mentioned intra­chain inter­actions [N1—H1⋯O7(Cl2)(*x* − 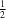, −*y* + 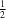, *z*) and N5—H5⋯O14(Cl3)(*x* − 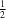, −*y* + 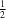, *z*)] results in the formation of sheets lying parallel to the (001) plane (Fig. 2 ▸), with a distance between them of 6.82 Å. There are also numerous intra- and inter­chain C—H⋯O contacts between methyl­ene groups of the macrocycle and the O atoms of the anions (Table 2 ▸), and these latter inter­actions are responsible for the formation of the three-dimensional structure of (I).

## Database survey   

A search of the Cambridge Structural Database (CSD, Version 5.40, last update February 2019; Groom *et al.*, 2016 ▸) indicated that among 76 Cu^II^ com­plexes of *N* ^3^,*N* ^10^-disubstituted di­aza­cyclam ligands, 37 com­pounds are formed by the ligands bearing alkyl substituents decorated with potentially coordinating groups (hy­droxy, imidazolyl, thienyl, amine, nitrile or carbox­yl) and many of them were investigated as building blocks for the construction of MOFs by using additional carboxyl­ate or metalocyanide linkers. At the same time, there are only two examples demonstrating self-polymerization (coordination of the substituent in the macrocycle with a neighbouring metal ion), namely, those with di­aza­cyclam ligands containing 2-propio­nitrile (refcode CAGHOI; Liu *et al.*, 2002 ▸) or 3-carb­oxy­propyl (WAMWIR; Ou *et al.*, 2005 ▸) donor groups. Among the Cu^II^ com­plexes of *N* ^3^-substituted aza­cyclam ligands only one com­plex with the 3-picolyl substituent that is potentially able to coordinate has been described (NOLDAW; Andriichuk *et al.*, 2019 ▸), thus the title com­pound (I) is the second example of a [Cu(aza­cyclam)]^2+^ cation of this kind described so far.

## Synthesis and crystallization   

All chemicals and solvents used in this work were purchased from Sigma–Aldrich and were used without further purification. The starting Cu^II^ com­plexes with an open-chain tetra­amine, [Cu(*2,3,2-tet*)](ClO_4_)_2_ (*2,3,2-tet* = 3,7-di­aza­nonane-1,9-di­amine), was prepared according to a published method (Maloshtan & Lampeka, 1996 ▸). Compound (I) was prepared as follows. A mixture of [Cu(*2,3,2-tet*)](ClO_4_)_2_ (200 mg, 0.46 mmol), 4-amino­butanoic acid (49 mg, 0.47 mmol) and 30% aqueous formaldehyde (0.24 ml, 3.2 mmol) in methanol (40 ml) was refluxed for 24 h. After cooling and filtration, the solution was kept in a refrigerator overnight. The violet crystalline precipitate was filtered off, washed with methanol (5 ml) and recrystallized from a 1:1 (*v*/*v*) water–ethanol solvent mixture (10 ml) containing 0.5 *M* perchloric acid (yield 84 mg, 28%). Analysis calculated (%) for C_13_H_30_Cl_3_CuN_5_O_14_: C 24.01, H 4.65, N 10.76; found: C 24.17, H 4.51, N 10.92. Violet blocks of (I) suitable for X-ray diffraction analysis were selected from the sample resulting from the synthesis.

**Safety note**: per­chlorate salts of metal com­plexes are potentially explosive and should be handled with care.

## Refinement   

Crystal data, data collection and structure refinement details are summarized in Table 3 ▸. All H atoms in (I) were placed in geometrically idealized positions and constrained to ride on their parent atoms, with C—H = 0.99 Å, N—H = 1.00 Å and carboxyl­ate O—H = 0.84 Å, with *U*_iso_(H) values of 1.2 or 1.5*U*_eq_ of the parent atoms. The crystal of (I) chosen for data collection was found to crystallize as an inversion twin.

## Supplementary Material

Crystal structure: contains datablock(s) I, global. DOI: 10.1107/S205698901901377X/hb7857sup1.cif

Structure factors: contains datablock(s) I. DOI: 10.1107/S205698901901377X/hb7857Isup2.hkl

CCDC references: 1958285, 1958285

Additional supporting information:  crystallographic information; 3D view; checkCIF report

## Figures and Tables

**Table 1:** Selected bond lengths and angles (Å, °)

Distances		Bite angles		
Cu1—N1	2.005 (19)	N1—Cu1—N2	84.4 (9)	
Cu1—N2	1.99 (2)	N4—Cu1—N5	88.0 (9)	
Cu1—N4	2.071 (19)	N1—Cu1—N5	93.9 (4)	
Cu1—N5	1.99 (2)	N2—Cu1—N4	93.4 (4)	
Cu1—O2	2.379 (8)			
Cu1—O3	2.544 (16)			
Cu1—O3*X*	2.687 (18)			

**Table 2:** Hydrogen-bond geometry (Å, °)

*D*—H⋯*A*	*D*—H	H⋯*A*	*D*⋯*A*	*D*—H⋯*A*
N2—H2⋯O8	1.00	2.35	3.15 (3)	136
N3—H3⋯O10	1.00	2.23	2.85 (2)	119
N3—H3⋯O12	1.00	2.43	2.99 (2)	115
N4—H4⋯O11	1.00	2.23	3.08 (3)	143
O1—H1*C*⋯O7^i^	0.84	2.39	3.13 (4)	147
N2—H2⋯O1^ii^	1.00	2.47	3.20 (3)	129
N4—H4⋯O1^ii^	1.00	2.43	3.13 (3)	126
N1—H1⋯O7^iii^	1.00	2.40	3.29 (3)	148
N5—H5⋯O14^iii^	1.00	2.35	3.26 (3)	151
C2—H2*A*⋯O3^iii^	0.99	2.63	3.15 (4)	113
C2—H2*B*⋯O8	0.99	2.64	3.26 (4)	120
C3—H3*A*⋯O3^iii^	0.99	2.65	3.23 (3)	118
C3—H3*B*⋯O11^iv^	0.99	2.57	3.42 (3)	144
C4—H4*A*⋯O4^iii^	0.99	2.44	3.40 (3)	163
C4—H4*B*⋯O8^v^	0.99	2.61	3.48 (3)	147
C5—H5*B*⋯O5^v^	0.99	2.65	3.29 (3)	122
C7—H7*A*⋯O2^ii^	0.99	2.64	3.23 (3)	118
C7—H7*A*⋯O4^vi^	0.99	2.65	3.26 (3)	120
C7—H7*A*⋯O9^vii^	0.99	2.64	3.44 (3)	138
C8—H8*B*⋯O5^vi^	0.99	2.65	3.56 (3)	153
C9—H9*B*⋯O2^ii^	0.99	2.58	3.20 (3)	120
C10—H10*B*⋯O10	0.99	2.60	3.15 (3)	115
C10—H10*A*⋯O12	0.99	2.56	3.18 (3)	121
C11—H11*B*⋯O9^v^	0.99	2.47	3.40 (4)	157
C11—H11*A*⋯O13^iv^	0.99	2.44	3.43 (3)	172
N1—H1⋯O6*X* ^iii^	1.00	2.46	3.36 (3)	149
N5—H5⋯O3*X* ^iii^	1.00	2.36	2.88 (3)	112
C3—H3*A*⋯O4*X* ^iii^	0.99	2.52	3.45 (4)	156
C4—H4*A*⋯O3*X* ^iii^	0.99	2.47	3.14 (3)	125
C5—H5*A*⋯O3*X* ^iii^	0.99	2.13	2.83 (4)	127
C6—H6*B*⋯O4*X* ^v^	0.99	2.61	3.48 (4)	147
C8—H8*B*⋯O5*X* ^vi^	0.99	2.65	3.59 (3)	157
C12—H12*B*⋯O5*X* ^i^	0.99	2.62	3.19 (3)	117
C12—H12*A*⋯O6*X* ^i^	0.99	2.52	3.25 (3)	130

Symmetry codes: (i) 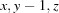; (ii) 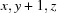; (iii) 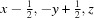; (iv) 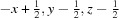; (v) 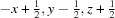; (vi) 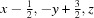; (vii) 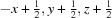.

**Table 3:** Experimental details

Crystal data	
Chemical formula	[Cu(C_13_H_30_N_5_O_2_)(ClO_4_)](ClO_4_)_2_
*M*_r_	650.31
Crystal system, space group	Orthorhombic, *P* *n* *a*2_1_
Temperature (K)	100
*a*, *b*, *c* (Å)	18.990 (4), 9.3640 (19), 13.636 (3)
*V* (Å^3^)	2424.9 (8)
*Z*	4
Radiation type	Mo *K*α
μ (mm^−1^)	1.31
Crystal size (mm)	0.18 × 0.14 × 0.12

Data collection	
Diffractometer	Bruker X8 APEXII CCD
Absorption correction	Multi-scan (*SADABS*; Bruker, 2007 ▸)
*T*_min_, *T*_max_	0.799, 0.859
No. of measured, independent and observed [*I* > 2σ(*I*)] reflections	60778, 4458, 2787
*R*_int_	0.150
(sin θ/λ)_max_ (Å^−1^)	0.604

Refinement	
*R*[*F* ^2^ > 2σ(*F* ^2^)], *wR*(*F* ^2^), *S*	0.070, 0.215, 1.03
No. of reflections	4458
No. of parameters	315
No. of restraints	161
H-atom treatment	H-atom parameters constrained
Δρ_max_, Δρ_min_ (e Å^−3^)	1.06, −1.45
Absolute structure	Refined as an inversion twin
Absolute structure parameter	0.50 (9)

Computer programs: *APEX2* (Bruker, 2007 ▸), *SAINT* (Bruker, 2007 ▸), *SHELXS2014* (Sheldrick, 2015*a* ▸), *SHELXL2014* (Sheldrick, 2015*b* ▸), *Mercury* (Macrae *et al.*, 2008 ▸) and *publCIF* (Westrip, 2010 ▸).

## supplementary crystallographic information

### Crystal data

**Table:**

[Cu(C_13_H_30_N_5_O_2_)(ClO_4_)](ClO_4_)_2_	*D*_x_ = 1.781 Mg m^−^^3^
*M**_r_* = 650.31	Mo *K*α radiation, λ = 0.71073 Å
Orthorhombic, *P**n**a*2_1_	Cell parameters from 4345 reflections
*a* = 18.990 (4) Å	θ = 2.1–24.5°
*b* = 9.3640 (19) Å	µ = 1.31 mm^−^^1^
*c* = 13.636 (3) Å	*T* = 100 K
*V* = 2424.9 (8) Å^3^	Block, violet
*Z* = 4	0.18 × 0.14 × 0.12 mm
*F*(000) = 1340	

### Data collection

**Table:**

Bruker X8 APEXII CCD diffractometer	2787 reflections with *I* > 2σ(*I*)
Radiation source: fine-focus sealed tube	*R*_int_ = 0.150
φ and ω scans	θ_max_ = 25.4°, θ_min_ = 2.2°
Absorption correction: multi-scan (SADABS; Bruker, 2007)	*h* = −22→22
*T*_min_ = 0.799, *T*_max_ = 0.859	*k* = −11→11
60778 measured reflections	*l* = −16→16
4458 independent reflections	

### Refinement

**Table:**

Refinement on *F*^2^	Hydrogen site location: inferred from neighbouring sites
Least-squares matrix: full	H-atom parameters constrained
*R*[*F*^2^ > 2σ(*F*^2^)] = 0.070	*w* = 1/[σ^2^(*F*_o_^2^) + (0.1002*P*)^2^ + 10.5107*P*] where *P* = (*F*_o_^2^ + 2*F*_c_^2^)/3
*wR*(*F*^2^) = 0.215	(Δ/σ)_max_ < 0.001
*S* = 1.03	Δρ_max_ = 1.06 e Å^−^^3^
4458 reflections	Δρ_min_ = −1.44 e Å^−^^3^
315 parameters	Absolute structure: Refined as an inversion twin
161 restraints	Absolute structure parameter: 0.50 (9)

### Special details

**Table:**

Geometry. All esds (except the esd in the dihedral angle between two l.s. planes) are estimated using the full covariance matrix. The cell esds are taken into account individually in the estimation of esds in distances, angles and torsion angles; correlations between esds in cell parameters are only used when they are defined by crystal symmetry. An approximate (isotropic) treatment of cell esds is used for estimating esds involving l.s. planes.
Refinement. Refined as a 2-component inversion twin

### Fractional atomic coordinates and isotropic or equivalent isotropic displacement parameters (Å^2^)

**Table:**

	*x*	*y*	*z*	*U*_iso_*/*U*_eq_	Occ. (<1)
Cu1	0.08430 (6)	0.21078 (13)	0.8220 (4)	0.0290 (4)	
Cl3	0.3273 (4)	0.1883 (8)	1.0262 (4)	0.068 (3)	
Cl2	0.3260 (4)	0.1803 (8)	0.6130 (4)	0.059 (2)	
O7	0.3630 (8)	0.3036 (16)	0.6503 (11)	0.068 (3)	0.8
O8	0.2596 (8)	0.224 (2)	0.5737 (13)	0.068 (3)	0.8
O9	0.3677 (8)	0.1169 (16)	0.5374 (11)	0.068 (3)	0.8
O10	0.3152 (10)	0.0807 (18)	0.6902 (10)	0.068 (3)	0.8
O7X	0.3906 (14)	0.238 (5)	0.578 (3)	0.068 (3)	0.2
O8X	0.275 (2)	0.292 (4)	0.622 (4)	0.068 (3)	0.2
O9X	0.300 (3)	0.076 (4)	0.543 (3)	0.068 (3)	0.2
O10X	0.335 (2)	0.112 (5)	0.7053 (19)	0.068 (3)	0.2
O11	0.2616 (8)	0.2416 (19)	1.0611 (13)	0.067 (3)	0.78
O12	0.3169 (9)	0.0676 (17)	0.9647 (11)	0.067 (3)	0.78
O13	0.3769 (7)	0.1662 (17)	1.1023 (10)	0.067 (3)	0.78
O14	0.3598 (8)	0.2996 (16)	0.9616 (10)	0.067 (3)	0.78
O11X	0.2692 (17)	0.281 (4)	1.051 (3)	0.067 (3)	0.22
O12X	0.311 (2)	0.114 (4)	0.937 (2)	0.067 (3)	0.22
O13X	0.336 (2)	0.085 (4)	1.104 (2)	0.067 (3)	0.22
O14X	0.3902 (16)	0.271 (4)	1.016 (3)	0.067 (3)	0.22
N4	0.1446 (10)	0.116 (2)	0.9306 (14)	0.028 (4)	
H4	0.1868	0.1769	0.9436	0.033*	
N5	0.0237 (12)	0.291 (2)	0.9273 (17)	0.039 (6)	
H5	−0.0196	0.2300	0.9302	0.047*	
N1	0.0240 (11)	0.2873 (19)	0.7134 (16)	0.030 (5)	
H1	−0.0194	0.2268	0.7106	0.036*	
N2	0.1386 (11)	0.1191 (19)	0.7138 (16)	0.031 (5)	
H2	0.1800	0.1829	0.7023	0.038*	
N3	0.2112 (4)	−0.0353 (9)	0.8184 (18)	0.027 (2)	
H3	0.2441	0.0479	0.8184	0.033*	
C5	0.0944 (15)	0.124 (3)	1.0203 (19)	0.041 (7)	
H5A	0.0566	0.0512	1.0139	0.049*	
H5B	0.1209	0.1040	1.0814	0.049*	
C6	0.0638 (16)	0.267 (3)	1.0239 (17)	0.042 (7)	
H6A	0.1014	0.3393	1.0312	0.051*	
H6B	0.0313	0.2752	1.0803	0.051*	
C7	−0.0001 (15)	0.442 (3)	0.914 (2)	0.039 (6)	
H7A	0.0413	0.5058	0.9123	0.046*	
H7B	−0.0304	0.4701	0.9694	0.046*	
C8	−0.0406 (6)	0.4551 (12)	0.819 (3)	0.040 (3)	
H8A	−0.0790	0.3834	0.8197	0.047*	
H8B	−0.0629	0.5506	0.8177	0.047*	
C9	0.0008 (17)	0.436 (3)	0.726 (2)	0.044 (7)	
H9A	−0.0287	0.4647	0.6695	0.053*	
H9B	0.0425	0.4997	0.7277	0.053*	
C1	0.0607 (16)	0.268 (3)	0.622 (2)	0.051 (9)	
H1A	0.0951	0.3467	0.6126	0.061*	
H1B	0.0268	0.2706	0.5668	0.061*	
C2	0.0999 (17)	0.121 (3)	0.625 (2)	0.045 (8)	
H2A	0.0656	0.0410	0.6228	0.054*	
H2B	0.1320	0.1114	0.5677	0.054*	
C3	0.1686 (14)	−0.026 (3)	0.728 (2)	0.036 (6)	
H3A	0.1297	−0.0967	0.7319	0.043*	
H3B	0.1983	−0.0513	0.6711	0.043*	
C4	0.1670 (14)	−0.027 (2)	0.9140 (17)	0.031 (6)	
H4A	0.1254	−0.0903	0.9083	0.037*	
H4B	0.1957	−0.0603	0.9701	0.037*	
C10	0.2553 (5)	−0.1691 (11)	0.821 (3)	0.034 (3)	
H10A	0.2849	−0.1678	0.8808	0.041*	
H10B	0.2871	−0.1697	0.7635	0.041*	
C11	0.2116 (6)	−0.3044 (11)	0.820 (3)	0.037 (3)	
H11A	0.1824	−0.3073	0.7604	0.045*	
H11B	0.1797	−0.3048	0.8778	0.045*	
Cl1	0.4733 (6)	0.6298 (13)	0.8275 (11)	0.049 (2)	0.5
O5	0.4266 (13)	0.681 (3)	0.7565 (17)	0.092 (3)	0.5
O4	0.5273 (11)	0.731 (2)	0.8438 (19)	0.092 (3)	0.5
O3	0.5054 (7)	0.5048 (18)	0.7937 (18)	0.092 (3)	0.5
O6	0.4378 (13)	0.605 (2)	0.9148 (16)	0.092 (3)	0.5
Cl1X	0.4594 (6)	0.5922 (13)	0.8170 (11)	0.049 (2)	0.5
O5X	0.4124 (13)	0.682 (3)	0.8729 (18)	0.092 (3)	0.5
O6X	0.4188 (12)	0.494 (2)	0.7562 (18)	0.092 (3)	0.5
O3X	0.5033 (7)	0.507 (2)	0.8825 (18)	0.092 (3)	0.5
O4X	0.5041 (13)	0.679 (2)	0.7532 (18)	0.092 (3)	0.5
O2	0.1552 (4)	−0.5798 (9)	0.819 (2)	0.052 (2)	
O1	0.2542 (5)	−0.6903 (9)	0.823 (3)	0.085 (5)	
H1C	0.2935	−0.6775	0.7958	0.128*	
C13	0.2181 (6)	−0.5702 (11)	0.821 (3)	0.037 (3)	
C12	0.2587 (6)	−0.4350 (12)	0.824 (3)	0.041 (3)	
H12A	0.2915	−0.4324	0.7675	0.049*	
H12B	0.2871	−0.4323	0.8847	0.049*	

### Atomic displacement parameters (Å^2^)

**Table:**

	*U*^11^	*U*^22^	*U*^33^	*U*^12^	*U*^13^	*U*^23^
Cu1	0.0320 (7)	0.0255 (7)	0.0296 (7)	0.0037 (5)	0.0012 (18)	0.0012 (18)
Cl3	0.045 (5)	0.090 (6)	0.069 (5)	0.014 (5)	−0.011 (4)	−0.046 (5)
Cl2	0.050 (5)	0.071 (4)	0.057 (5)	0.008 (4)	0.009 (4)	0.027 (4)
O7	0.060 (7)	0.072 (7)	0.071 (6)	0.002 (5)	0.015 (5)	0.023 (5)
O8	0.060 (7)	0.072 (7)	0.071 (6)	0.002 (5)	0.015 (5)	0.023 (5)
O9	0.060 (7)	0.072 (7)	0.071 (6)	0.002 (5)	0.015 (5)	0.023 (5)
O10	0.060 (7)	0.072 (7)	0.071 (6)	0.002 (5)	0.015 (5)	0.023 (5)
O7X	0.060 (7)	0.072 (7)	0.071 (6)	0.002 (5)	0.015 (5)	0.023 (5)
O8X	0.060 (7)	0.072 (7)	0.071 (6)	0.002 (5)	0.015 (5)	0.023 (5)
O9X	0.060 (7)	0.072 (7)	0.071 (6)	0.002 (5)	0.015 (5)	0.023 (5)
O10X	0.060 (7)	0.072 (7)	0.071 (6)	0.002 (5)	0.015 (5)	0.023 (5)
O11	0.046 (5)	0.080 (7)	0.074 (6)	−0.006 (5)	0.004 (4)	−0.037 (5)
O12	0.046 (5)	0.080 (7)	0.074 (6)	−0.006 (5)	0.004 (4)	−0.037 (5)
O13	0.046 (5)	0.080 (7)	0.074 (6)	−0.006 (5)	0.004 (4)	−0.037 (5)
O14	0.046 (5)	0.080 (7)	0.074 (6)	−0.006 (5)	0.004 (4)	−0.037 (5)
O11X	0.046 (5)	0.080 (7)	0.074 (6)	−0.006 (5)	0.004 (4)	−0.037 (5)
O12X	0.046 (5)	0.080 (7)	0.074 (6)	−0.006 (5)	0.004 (4)	−0.037 (5)
O13X	0.046 (5)	0.080 (7)	0.074 (6)	−0.006 (5)	0.004 (4)	−0.037 (5)
O14X	0.046 (5)	0.080 (7)	0.074 (6)	−0.006 (5)	0.004 (4)	−0.037 (5)
N4	0.025 (9)	0.038 (11)	0.020 (8)	0.006 (9)	−0.003 (8)	−0.003 (8)
N5	0.033 (13)	0.046 (17)	0.039 (12)	0.002 (10)	−0.007 (11)	0.009 (11)
N1	0.031 (13)	0.017 (12)	0.041 (12)	0.007 (8)	−0.009 (10)	0.006 (9)
N2	0.034 (11)	0.017 (9)	0.043 (11)	−0.004 (8)	−0.007 (9)	−0.004 (9)
N3	0.027 (4)	0.024 (5)	0.031 (5)	0.000 (4)	−0.002 (11)	0.010 (11)
C5	0.053 (16)	0.048 (17)	0.022 (14)	0.013 (13)	0.016 (11)	0.004 (12)
C6	0.064 (19)	0.048 (17)	0.015 (11)	0.004 (14)	−0.005 (11)	−0.012 (10)
C7	0.036 (15)	0.026 (14)	0.054 (16)	0.000 (13)	0.009 (13)	−0.015 (12)
C8	0.033 (6)	0.028 (6)	0.057 (8)	0.011 (5)	−0.008 (17)	0.015 (16)
C9	0.040 (17)	0.032 (15)	0.061 (17)	0.008 (14)	−0.005 (15)	−0.001 (13)
C1	0.042 (16)	0.046 (17)	0.06 (2)	0.020 (13)	−0.021 (14)	−0.008 (14)
C2	0.052 (16)	0.043 (17)	0.039 (18)	0.021 (13)	−0.003 (12)	−0.006 (14)
C3	0.033 (15)	0.031 (14)	0.044 (15)	0.001 (13)	0.000 (12)	−0.003 (12)
C4	0.036 (15)	0.026 (14)	0.030 (13)	0.010 (13)	0.009 (11)	0.007 (11)
C10	0.029 (5)	0.022 (5)	0.052 (7)	0.007 (4)	0.015 (16)	0.002 (17)
C11	0.035 (6)	0.027 (6)	0.050 (7)	−0.001 (5)	−0.006 (17)	−0.017 (15)
Cl1	0.045 (4)	0.057 (6)	0.045 (3)	0.018 (4)	−0.009 (6)	−0.015 (7)
O5	0.089 (6)	0.080 (7)	0.106 (9)	0.026 (6)	0.000 (8)	−0.004 (7)
O4	0.089 (6)	0.080 (7)	0.106 (9)	0.026 (6)	0.000 (8)	−0.004 (7)
O3	0.089 (6)	0.080 (7)	0.106 (9)	0.026 (6)	0.000 (8)	−0.004 (7)
O6	0.089 (6)	0.080 (7)	0.106 (9)	0.026 (6)	0.000 (8)	−0.004 (7)
Cl1X	0.045 (4)	0.057 (6)	0.045 (3)	0.018 (4)	−0.009 (6)	−0.015 (7)
O5X	0.089 (6)	0.080 (7)	0.106 (9)	0.026 (6)	0.000 (8)	−0.004 (7)
O6X	0.089 (6)	0.080 (7)	0.106 (9)	0.026 (6)	0.000 (8)	−0.004 (7)
O3X	0.089 (6)	0.080 (7)	0.106 (9)	0.026 (6)	0.000 (8)	−0.004 (7)
O4X	0.089 (6)	0.080 (7)	0.106 (9)	0.026 (6)	0.000 (8)	−0.004 (7)
O2	0.029 (4)	0.032 (5)	0.093 (7)	−0.006 (4)	−0.010 (14)	0.007 (13)
O1	0.046 (5)	0.026 (5)	0.184 (14)	0.005 (4)	−0.035 (18)	0.014 (17)
C13	0.044 (7)	0.020 (6)	0.045 (7)	0.003 (5)	0.018 (16)	0.013 (15)
C12	0.025 (6)	0.035 (6)	0.063 (8)	0.002 (5)	0.017 (16)	0.008 (17)

### Geometric parameters (Å, º)

**Table:**

Cu1—N5	1.99 (2)	C6—H6A	0.9900
Cu1—N2	1.99 (2)	C6—H6B	0.9900
Cu1—N1	2.005 (19)	C7—C8	1.51 (4)
Cu1—N4	2.071 (19)	C7—H7A	0.9900
Cu1—O2^i^	2.379 (8)	C7—H7B	0.9900
Cl3—O13	1.417 (12)	C8—C9	1.50 (5)
Cl3—O12	1.422 (12)	C8—H8A	0.9900
Cl3—O11	1.426 (12)	C8—H8B	0.9900
Cl3—O14X	1.429 (15)	C9—H9A	0.9900
Cl3—O12X	1.435 (15)	C9—H9B	0.9900
Cl3—O11X	1.446 (14)	C1—C2	1.57 (4)
Cl3—O13X	1.447 (15)	C1—H1A	0.9900
Cl3—O14	1.497 (13)	C1—H1B	0.9900
Cl2—O10	1.421 (13)	C2—H2A	0.9900
Cl2—O7X	1.424 (15)	C2—H2B	0.9900
Cl2—O10X	1.424 (15)	C3—H3A	0.9900
Cl2—O9	1.430 (13)	C3—H3B	0.9900
Cl2—O8	1.431 (13)	C4—H4A	0.9900
Cl2—O8X	1.437 (15)	C4—H4B	0.9900
Cl2—O7	1.443 (13)	C10—C11	1.514 (14)
Cl2—O9X	1.448 (15)	C10—H10A	0.9900
N4—C4	1.42 (3)	C10—H10B	0.9900
N4—C5	1.55 (3)	C11—C12	1.515 (15)
N4—H4	1.0000	C11—H11A	0.9900
N5—C7	1.50 (3)	C11—H11B	0.9900
N5—C6	1.54 (3)	Cl1—O6	1.387 (17)
N5—H5	1.0000	Cl1—O3	1.398 (17)
N1—C1	1.44 (4)	Cl1—O5	1.399 (17)
N1—C9	1.47 (3)	Cl1—O4	1.411 (17)
N1—H1	1.0000	Cl1X—O5X	1.446 (19)
N2—C2	1.42 (3)	Cl1X—O3X	1.456 (19)
N2—C3	1.49 (3)	Cl1X—O6X	1.461 (19)
N2—H2	1.0000	Cl1X—O4X	1.463 (19)
N3—C3	1.47 (3)	O2—C13	1.198 (13)
N3—C10	1.507 (13)	O2—Cu1^ii^	2.379 (8)
N3—C4	1.55 (3)	O1—C13	1.318 (13)
N3—H3	1.0000	O1—H1C	0.8400
C5—C6	1.46 (4)	C13—C12	1.483 (16)
C5—H5A	0.9900	C12—H12A	0.9900
C5—H5B	0.9900	C12—H12B	0.9900
			
N5—Cu1—N2	175.2 (9)	N5—C7—H7A	109.7
N5—Cu1—N1	93.9 (4)	C8—C7—H7A	109.7
N2—Cu1—N1	84.4 (9)	N5—C7—H7B	109.7
N5—Cu1—N4	88.0 (9)	C8—C7—H7B	109.7
N2—Cu1—N4	93.4 (4)	H7A—C7—H7B	108.2
N1—Cu1—N4	175.6 (8)	C9—C8—C7	116.4 (10)
N5—Cu1—O2^i^	91.8 (9)	C9—C8—H8A	108.2
N2—Cu1—O2^i^	92.8 (8)	C7—C8—H8A	108.2
N1—Cu1—O2^i^	90.8 (8)	C9—C8—H8B	108.2
N4—Cu1—O2^i^	93.1 (8)	C7—C8—H8B	108.2
O13—Cl3—O12	114.1 (7)	H8A—C8—H8B	107.3
O13—Cl3—O11	112.8 (7)	N1—C9—C8	112 (2)
O12—Cl3—O11	110.7 (7)	N1—C9—H9A	109.3
O14X—Cl3—O12X	110.8 (9)	C8—C9—H9A	109.3
O14X—Cl3—O11X	109.5 (9)	N1—C9—H9B	109.3
O12X—Cl3—O11X	109.1 (9)	C8—C9—H9B	109.3
O14X—Cl3—O13X	109.6 (9)	H9A—C9—H9B	108.0
O12X—Cl3—O13X	109.0 (9)	N1—C1—C2	109 (2)
O11X—Cl3—O13X	108.7 (9)	N1—C1—H1A	110.0
O13—Cl3—O14	105.1 (7)	C2—C1—H1A	110.0
O12—Cl3—O14	105.3 (7)	N1—C1—H1B	110.0
O11—Cl3—O14	108.3 (7)	C2—C1—H1B	110.0
O7X—Cl2—O10X	111.1 (9)	H1A—C1—H1B	108.3
O10—Cl2—O9	110.0 (7)	N2—C2—C1	106 (2)
O10—Cl2—O8	109.8 (7)	N2—C2—H2A	110.6
O9—Cl2—O8	109.7 (7)	C1—C2—H2A	110.6
O7X—Cl2—O8X	109.7 (9)	N2—C2—H2B	110.6
O10X—Cl2—O8X	110.0 (9)	C1—C2—H2B	110.6
O10—Cl2—O7	109.5 (7)	H2A—C2—H2B	108.7
O9—Cl2—O7	108.5 (7)	N3—C3—N2	111.8 (18)
O8—Cl2—O7	109.4 (7)	N3—C3—H3A	109.2
O7X—Cl2—O9X	108.9 (9)	N2—C3—H3A	109.2
O10X—Cl2—O9X	108.6 (9)	N3—C3—H3B	109.2
O8X—Cl2—O9X	108.4 (9)	N2—C3—H3B	109.2
C4—N4—C5	110.6 (19)	H3A—C3—H3B	107.9
C4—N4—Cu1	117.0 (15)	N4—C4—N3	110.0 (17)
C5—N4—Cu1	101.8 (14)	N4—C4—H4A	109.7
C4—N4—H4	109.0	N3—C4—H4A	109.7
C5—N4—H4	109.0	N4—C4—H4B	109.7
Cu1—N4—H4	109.0	N3—C4—H4B	109.7
C7—N5—C6	113 (2)	H4A—C4—H4B	108.2
C7—N5—Cu1	116.2 (19)	N3—C10—C11	113.0 (8)
C6—N5—Cu1	106.1 (15)	N3—C10—H10A	109.0
C7—N5—H5	107.0	C11—C10—H10A	109.0
C6—N5—H5	107.0	N3—C10—H10B	109.0
Cu1—N5—H5	107.0	C11—C10—H10B	109.0
C1—N1—C9	111 (2)	H10A—C10—H10B	107.8
C1—N1—Cu1	108.6 (16)	C10—C11—C12	110.6 (9)
C9—N1—Cu1	115.0 (19)	C10—C11—H11A	109.5
C1—N1—H1	107.2	C12—C11—H11A	109.5
C9—N1—H1	107.2	C10—C11—H11B	109.5
Cu1—N1—H1	107.2	C12—C11—H11B	109.5
C2—N2—C3	108.6 (19)	H11A—C11—H11B	108.1
C2—N2—Cu1	111.3 (16)	O6—Cl1—O3	110.9 (9)
C3—N2—Cu1	119.6 (17)	O6—Cl1—O5	110.0 (8)
C2—N2—H2	105.4	O3—Cl1—O5	109.7 (9)
C3—N2—H2	105.4	O6—Cl1—O4	109.2 (9)
Cu1—N2—H2	105.4	O3—Cl1—O4	107.1 (8)
C3—N3—C10	112 (2)	O5—Cl1—O4	109.8 (8)
C3—N3—C4	113.6 (8)	O5X—Cl1X—O3X	110.4 (8)
C10—N3—C4	109 (2)	O5X—Cl1X—O6X	110.0 (8)
C3—N3—H3	107.4	O3X—Cl1X—O6X	107.8 (8)
C10—N3—H3	107.4	O5X—Cl1X—O4X	110.3 (8)
C4—N3—H3	107.4	O3X—Cl1X—O4X	109.7 (8)
C6—C5—N4	108 (2)	O6X—Cl1X—O4X	108.7 (8)
C6—C5—H5A	110.1	C13—O2—Cu1^ii^	128.7 (7)
N4—C5—H5A	110.1	C13—O1—H1C	109.5
C6—C5—H5B	110.1	O2—C13—O1	117.1 (10)
N4—C5—H5B	110.1	O2—C13—C12	125.7 (10)
H5A—C5—H5B	108.4	O1—C13—C12	117.2 (10)
C5—C6—N5	108 (2)	C13—C12—C11	112.4 (9)
C5—C6—H6A	110.2	C13—C12—H12A	109.1
N5—C6—H6A	110.2	C11—C12—H12A	109.1
C5—C6—H6B	110.2	C13—C12—H12B	109.1
N5—C6—H6B	110.2	C11—C12—H12B	109.1
H6A—C6—H6B	108.5	H12A—C12—H12B	107.8
N5—C7—C8	110 (2)		
			
C4—N4—C5—C6	−171 (2)	C10—N3—C3—N2	−166.3 (16)
Cu1—N4—C5—C6	−45 (2)	C4—N3—C3—N2	70.0 (19)
N4—C5—C6—N5	60 (3)	C2—N2—C3—N3	178 (2)
C7—N5—C6—C5	−170 (2)	Cu1—N2—C3—N3	−52 (2)
Cu1—N5—C6—C5	−42 (3)	C5—N4—C4—N3	174.6 (18)
C6—N5—C7—C8	−178 (2)	Cu1—N4—C4—N3	59 (2)
Cu1—N5—C7—C8	58 (3)	C3—N3—C4—N4	−75.1 (19)
N5—C7—C8—C9	−68 (2)	C10—N3—C4—N4	159.7 (17)
C1—N1—C9—C8	177 (2)	C3—N3—C10—C11	−62 (3)
Cu1—N1—C9—C8	−59 (3)	C4—N3—C10—C11	64 (3)
C7—C8—C9—N1	69 (2)	N3—C10—C11—C12	−180 (3)
C9—N1—C1—C2	166 (2)	Cu1^ii^—O2—C13—O1	2 (6)
Cu1—N1—C1—C2	38 (3)	Cu1^ii^—O2—C13—C12	−177 (3)
C3—N2—C2—C1	172 (2)	O2—C13—C12—C11	−2 (6)
Cu1—N2—C2—C1	38 (3)	O1—C13—C12—C11	179 (3)
N1—C1—C2—N2	−51 (3)	C10—C11—C12—C13	−179 (3)

Symmetry codes: (i) *x*, *y*+1, *z*; (ii) *x*, *y*−1, *z*.

### Hydrogen-bond geometry (Å, º)

**Table:**

*D*—H···*A*	*D*—H	H···*A*	*D*···*A*	*D*—H···*A*
N2—H2···O8	1.00	2.35	3.15 (3)	136
N3—H3···O10	1.00	2.23	2.85 (2)	119
N3—H3···O12	1.00	2.43	2.99 (2)	115
N4—H4···O11	1.00	2.23	3.08 (3)	143
O1—H1*C*···O7^ii^	0.84	2.39	3.13 (4)	147
N2—H2···O1^i^	1.00	2.47	3.20 (3)	129
N4—H4···O1^i^	1.00	2.43	3.13 (3)	126
N1—H1···O7^iii^	1.00	2.40	3.29 (3)	148
N5—H5···O14^iii^	1.00	2.35	3.26 (3)	151
C2—H2*A*···O3^iii^	0.99	2.63	3.15 (4)	113
C2—H2*B*···O8	0.99	2.64	3.26 (4)	120
C3—H3*A*···O3^iii^	0.99	2.65	3.23 (3)	118
C3—H3*B*···O11^iv^	0.99	2.57	3.42 (3)	144
C4—H4*A*···O4^iii^	0.99	2.44	3.40 (3)	163
C4—H4*B*···O8^v^	0.99	2.61	3.48 (3)	147
C5—H5*B*···O5^v^	0.99	2.65	3.29 (3)	122
C7—H7*A*···O2^i^	0.99	2.64	3.23 (3)	118
C7—H7*A*···O4^vi^	0.99	2.65	3.26 (3)	120
C7—H7*A*···O9^vii^	0.99	2.64	3.44 (3)	138
C8—H8*B*···O5^vi^	0.99	2.65	3.56 (3)	153
C9—H9*B*···O2^i^	0.99	2.58	3.20 (3)	120
C10—H10*B*···O10	0.99	2.60	3.15 (3)	115
C10—H10*A*···O12	0.99	2.56	3.18 (3)	121
C11—H11*B*···O9^v^	0.99	2.47	3.40 (4)	157
C11—H11*A*···O13^iv^	0.99	2.44	3.43 (3)	172
N1—H1···O6*X*^iii^	1.00	2.46	3.36 (3)	149
N5—H5···O3*X*^iii^	1.00	2.36	2.88 (3)	112
C3—H3*A*···O4*X*^iii^	0.99	2.52	3.45 (4)	156
C4—H4*A*···O3*X*^iii^	0.99	2.47	3.14 (3)	125
C5—H5*A*···O3*X*^iii^	0.99	2.13	2.83 (4)	127
C6—H6*B*···O4*X*^v^	0.99	2.61	3.48 (4)	147
C8—H8*B*···O5*X*^vi^	0.99	2.65	3.59 (3)	157
C12—H12*B*···O5*X*^ii^	0.99	2.62	3.19 (3)	117
C12—H12*A*···O6*X*^ii^	0.99	2.52	3.25 (3)	130

Symmetry codes: (i) *x*, *y*+1, *z*; (ii) *x*, *y*−1, *z*; (iii) *x*−1/2, −*y*+1/2, *z*; (iv) −*x*+1/2, *y*−1/2, *z*−1/2; (v) −*x*+1/2, *y*−1/2, *z*+1/2; (vi) *x*−1/2, −*y*+3/2, *z*; (vii) −*x*+1/2, *y*+1/2, *z*+1/2.
